# Fighting SARS-CoV-2 with green seaweed *Ulva* sp. extract: extraction protocol predetermines crude ulvan extract anti-SARS-CoV-2 inhibition properties in *in vitro* Vero-E6 cells assay

**DOI:** 10.7717/peerj.12398

**Published:** 2021-11-15

**Authors:** Shai Shefer, Arthur Robin, Alexander Chemodanov, Mario Lebendiker, Robert Bostwick, Lynn Rasmussen, Michael Lishner, Michael Gozin, Alexander Golberg

**Affiliations:** 1 Port School of Environment and Earth Sciences, Tel Aviv University, Tel Aviv, Israel; 2 Silberman Institute of Life Science, Hebrew University of Jerusalem, Jeruslem, Israel; 3 Sothern Research, Birmingham, AL, United States of America; 4 Meir Medical Center, Kvar Sava, Israel; 5 School of Chemisty, Tel Aviv University, Tel Aviv, Israel

**Keywords:** SARS-CoV-2, Antiviral, Seaweed, Macroalgae, Sulfated polysaccharides, *Ulva* sp., Extraction, Ulvan

## Abstract

Due to the global COVID-19 pandemic, there is a need to screen for novel compounds with antiviral activity against SARS-COV-2. Here we compared chemical composition and the *in vitro* anti- SARS-COV-2 activity of two different *Ulva* sp. crude ulvan extracts: one obtained by an HCl-based and another one by ammonium oxalate-based (AOx) extraction protocols. The composition of the crude extracts was analyzed and their antiviral activity was assessed in a cytopathic effect reduction assay using Vero E6 cells. We show that the extraction protocols have a significant impact on the chemical composition, anti- SARS-COV-2 activity, and cytotoxicity of these ulvan extracts. The ulvan extract based on the AOx protocol had a higher average molecular weight, higher charge, and 11.3-fold higher antiviral activity than HCl-based extract. Our results strongly suggest that further bioassay-guided investigation into bioactivity of compounds found in *Ulva* sp. ulvan extracts could lead to the discovery of novel anti-SARS-CoV-2 antivirals.

## Introduction

The COVID-19 global pandemic is caused by SARS-COV-2 and its mutations, which infected more than 100 million and caused the death of more than 2 million people (World Health Organization, 21st January 2021). In addition to its fast propagation and lethality, COVID-19 is causing a profound worldwide disruption of social and economic activities (Nicola et al., 2020). Moreover, serious post-illness long-term health damages were reported in many COVID-19 recovered patients (Mitrani, Dabas & Goldberger, 2020; Balachandar et al., 2020). Whereas the administration of new vaccines was initiated at the end of 2020 as a preventive measure against the spreading of the infection, alarming reports are being released regarding the potential of mutant strains to jeopardize both vaccine-based immunity and immunity developed from previous infections with the initial strains (Baum et al., 2020; Nelson et al., 2021; Zhang et al., 2021; Wibmer et al., 2021). Presently, no broadly accepted and efficient anti-viral drug treatment for the COVID-19 has been implemented. Thus, the need of finding new antiviral candidates is crucial for the treatment of SARS-COV-2 (Hilgenfeld & Peiris, 2013).

A rich variety of synthetic compounds and biomolecules exhibit antiviral properties. Notably, a considerable amount of knowledge has been accumulated regarding antivirals that originated from terrestrial plants and organisms (Li et al., 2005; Hudson, 2018). In contrast, the number of published articles that focused on antiviral biomolecules that originated from the marine environment is at least two orders of magnitude smaller than from terrestrial ones (Li et al., 2005; Uzair, Mahmood & Tabassum, 2011; Donia & Hamann, 2003). Out of the rich marine fauna and flora, macroalgae (or seaweed) are a large group of multicellular organisms encompassing several thousands of species, ranging from microscopic to large specimens of up to 30 m in length. Despite their diversity, macroalgae are much less explored than terrestrial organisms, while potentially containing numerous novel antivirals (Luescher-Mattli, 2003; Pujol et al, 2007; Witvrouw & De Clercq, 1997).

One of the significant differences between macroalgae and terrestrial and freshwater plants is the composition of their cell wall. More specifically, macroalgae differ from terrestrial plants in the chemical composition of their structural polysaccharides, many of which are sulfated (Jiao et al., 2011). Although the exact role of these seaweed sulfated polysaccharides (SSPS) is not determined, it was suggested that they can play a role in ion exchange, nutrient binding, and concentration processes in the marine environment, as well as defense against pathogens and opportunistic organisms (Kloareg & Quatrano, 1988; Ingle et al., 2018; Alves, Sousa & Reis, 2013).

The inhibitory effects of SSPS on viral replication *in vitro* have been known for more than 60 years with activities against several enveloped RNA viruses, the family of the current source of pandemic SARS-CoV-2, such as HIV (various strains), Sindbis, Semliki forest, Junin, Tacaribe, VSV, Influenza A, and RSV viruses (Luescher-Mattli, 2003; Pujol et al, 2007; Witvrouw & De Clercq, 1997; Gerber et al, 1958; Chiu et al., 2012; Hayashi, Hamada & Hayashi, 1996; Lee et al., 2004; Besednova et al, 2016; Terasawa et al., 2020). As an example, SSPS was found to block HIV replication in cell cultures, at concentrations as low as 10 µg mL^−1^ and without showing any toxicity to the host cells at concentrations of up to 2.5 mg mL^−1^ (Luescher-Mattli, 2003; Witvrouw & De Clercq, 1997). The antiviral activities of such SSPS partially correlate with the presence of charged groups, mainly the sulfonate groups, which are present to a high degree in certain SSPS. However, the sulfonate group presence alone was not sufficient for explaining the antiviral activities of these compounds (Pujol et al, 2007; Hayashi, Hamada & Hayashi, 1996; Lee et al., 2004; Ghosh et al., 2009).

The interactions between virus envelope proteins and sulfated polymers are keys to the antiviral activity of the latter (Luescher-Mattli, 2003; Witvrouw & De Clercq, 1997; Ghosh et al., 2009; Jin et al., 2020; Chen et al., 2020b; Song et al., 2020). Besides a high charge density on the sulfated polymer, a high level of structural flexibility seems to be required for efficient binding of the polysaccharides to the protein located on the surface of viruses such as the dengue and SARS-COV2 viruses (Ghosh et al., 2009; Jin et al., 2020; Song et al., 2020; Damonte, Matulewicz & Cerezo, 2004; Marks et al., 2001).

Thus, the inhibitory activity of the polysaccharides against SARS-CoV-2 may be also influenced by their structure (Ghosh et al., 2009; Damonte, Matulewicz & Cerezo, 2004; Marks et al., 2001; Kwon et al., 2020). The diversity of SSPS structures and sulfonate group content could be the main attributes behind those biopolymers’ broad antiviral activity, which inspired several investigators to call for additional testing of SSPS against SARS-CoV2 (Chen et al., 2020b; Pereira & Critchley, 2020; Bhatt, Arora & Prajapati, 2020). Subsequently, iota-carrageenan and fucoidan SSPS, extracted from red and brown seaweeds, were found to have antiviral activity against SARS-CoV2, among other investigated sulfated polymers (Song et al., 2020; Kwon et al., 2020). Although sulfated rhamans, part of ulvan polymers, found in green seaweed, were shown to have antiviral effects against a previous strain of human viruses such as corona and influenza viruses (Lee et al., 2004; Terasawa et al., 2020; Kyoko, Toshimitsu & Jung-bum, 2006; Lopes et al., 2017), there is no report regarding ulvan activity against SARS-CoV2.

Here we report on the anti-SARS-CoV-2 activity of two crude ulvans, extracted from a cultivated green seaweed *Ulva* sp., in an *in vitro* cytopathic effect (CPE) reduction assay using Vero E6 cells that are expressing angiotensin-converting enzyme 2 (ACE2) receptor to which SARS-CoV-2 virus typically binds (Lan et al., 2020; Li et al., 2003). These two extracts of ulvan, a known SSPS of *Ulva* sp. green seaweed, were obtained by two different extraction protocols, namely an HCl- and an ammonium oxalate. Extraction protocols greatly affect the quantity and quality of the ulvan extracts, notably their purity, molecular weight distribution, sulfate content, and bioactivity (Kidgell et al., 2019). The advantage of using *Ulva* sp. is in the possibility of controlling SSPS content and composition under-regulated growth conditions (Robin et al., 2017; Chemodanov, Robin & Golberg, 2017). As presently, there is no accepted and efficient treatment for the SARS-COV-2, and there is still a need for new antiviral therapeutics, we believe this work provides essential information for further development of natural product-based therapeutic agents against SARS-CoV-2 and its mutants’ pandemics (Lahlou, 2013).

## Materials and Methods

### *Ulva sp*. biomass cultivation

Green macroalgae *Ulva* sp. was grown under controlled media from April to May 2020 and illumination conditions, using macroalgae photobioreactors (MPBR) (Chemodanov, Robin & Golberg, 2017). In terms of the taxonomic status, the used macroalgae contained a mixture of *Ulva rigida* and *Ulva lactuca (formally Ulva fasciata*) (Nimrod Krupnik et al., 2018). The growth media was based on Mediterranean seawater, to which ammonium nitrate (NH_4_NO_3_) and phosphoric acid (H_3_PO_4_) (Haifa Chemicals Ltd., Israel) were added, to adjust total nitrogen and phosphorous contents to 6.4 and 0.97 g m^−3^, respectively, and pH to 8.2. Throughout the biomass cultivation, agitation and CO_2_ were provided by air bubbling at a flow rate of 2–4 L min^−1^. Before further use, fresh *Ulva* sp. biomass was harvested, washed from minerals and epiphytes with deionized water, and centrifuged at 2,800 rpm to remove the excess water.

### Ulvan extraction

#### Extraction with Ammonium Oxalate (AOx Protocol)

A crude extraction of the ulvan from tap water washed *Ulva* sp. biomass was carried out according to a modified Robic’s protocol (Robic et al., 2009c). First, the biomass was ground with an addition of deionized water to form a wet homogeneous paste, from which surplus water was removed by squeezing it out in a filter bag. Then, from the resulted wet material, pigments were extracted with ethanol at room temperature. Following ethanol treatment, filtering, and air drying, ulvan was extracted for 2 h with a vigorously stirred hot solution (75 ± 5 °C) of aqueous ammonium oxalate ([NH_4_]_2_[C_2_O_4_], 20 mM, pH 7) (Alves, Sousa & Reis, 2013). After that time, the supernatant was collected by centrifugation and its volume was reduced by 90% on a rotary evaporator at 60 ± 5 °C/80 mbar. To obtain a dry extract, the concentrated supernatant solution was dialyzed against deionized water, using 3.5 kDa dialysis membranes for 24 hrs, and was subsequently lyophilized. The resulting dry ulvan powder was further sterilized with a low-intensity *γ*-irradiation treatment (25 kGy (Munarin et al., 2013), Soreq Nuclear Research Center, Israel) and kept at −20 °C before further use.

#### Extraction with aqueous hydrochloric acid (HCl Protocol)

The solid material resulting from ethanol treatment, filtering, and air drying was suspended in deionized water (1:30 w/v) and the pH of this suspension was adjusted to 2.0−2.5 (at room temperature) by dropwise addition of aqueous HCl (5%). As in the previous protocol, hot extraction (75 ± 5 °C) of ulvan was conducted for 2 h with vigorous stirring (inspired by Kidgell, 2019 Kidgell et al. (2019)). After that time, the resulted mixture was cooled down to room temperature, and solid residues were removed first by filtration through a “parachute silk” (woven nylon) filter and then by subsequent centrifugation at 1, 792 × g (15 min, 4 °C) (Yingtai Instruments, model TGL18, China). Subsequently, the supernatant was adjusted to a neutral pH by dropwise addition of aqueous NaOH (2.0 M), and the volume of the supernatant was reduced by 90% on a rotary evaporator at 60 ± 5 °C/80 mbar. As previously mentioned, the dry extract was obtained by dialysis of the concentrated supernatant against deionized water, followed by lyophilization. The resulting dry ulvan powder was sterilized by *γ*-irradiation and kept at −20 °C before further use.

### Elemental and FTIR analyses

Elemental analyses of solid freeze-dried ulvan extract samples were performed on a CHNS elemental analyzer (Flash2000, Thermo Scientific) at the Schulich Faculty of Chemistry of the Technion (Haifa, Israel). FT-IR spectroscopy analyses of the solid freeze-dried ulvan extract samples were performed on a spectrometer (Tensor 27, Bruker) equipped with a standard attenuated total reflection attachment (ATR, Pike). FT-IR spectra of the extracts were measured in the spectral range of 4,000–400 cm^−1^ (4 cm^−1^ resolution). Samples we analyzed at least in a duplicate.

### Size exclusion and anion exchange chromatography

#### Sample preparation for chromatography

A sample of solid ulvan extract (20 mg) was suspended in HPLC-grade water (20 mL) and kept for 10 min at RT. Then, the undissolved solids were removed by centrifugation (13,000 rpm) and the resulted supernatant was further filtered through a 0.22 µm filter (PVDF, Merck).

#### Size exclusion chromatography (SEC) analysis

SEC analyses were performed at room temperature on a system (AKTA Pure, Cytiva), equipped with Superose 6 Increase 10/300 GL column (23 ml, Cytiva) and with an array of detectors, comprised of UV-vis detector (UV-900, Cytiva), a multi-angle light scattering (MALS; miniDAWN TREOS, Wyatt Technology) detector, a dynamic light scattering module (WyattQELS) and with a refractive index detector (Optilab T-rEX, Wyatt Technology). The elution was monitored at 280, 260, and 220 nm (UV–vis detector) and three angles (43.6, 90, and 136.4°) with a 658.9 nm laser beam (MALS detector) (Amartely et al., 2019). The refractive index of the eluting solvent was determined to be 1.331, the viscosity was 0.8945 cP (typical for the PBS buffer) and the refractive index increment value (d_n_/d_c_) for ulvan was defined to be 0.127 mL g^−1^ (Paradossi, Cavalieri & Chiessi, 2002). The data collection and analyses were performed with ASTRA 6.1 software (Wyatt Technology). A sample (with a typical volume of 0.5 mL) was injected into the column equilibrated with an eluent comprised of MES buffer (10 mM, pH 6.0) and NaCl (50 mM). A typical flow rate was 0.8 mL min^−1^ and for molecular weight (MW) calibration of this column see Table S1.

#### Anion exclusion chromatography (AEX) analysis

AEX analyses were performed at room temperature on the aforementioned system (AKTA Pure, Cytiva) equipped with a high resolution, anion exchange Mono-Q HR 5/5 column (one mL, Cytiva) and with the same array of detectors (Amartely et al., 2019; Amartely et al., 2018). For AEX analysis, a sample of PVDF filtered extract (0.5 mL) was diluted ten-fold with eluent A (sodium acetate buffer, 20 mM, pH 5.0) and injected (five mL) into the Mono-Q HR 5/5 column equilibrated with the eluent A. Typical separation conditions included a gradient elution (with a flow rate of 1.5 mL min^−1^) starting with an eluent A and gradual increase in sodium chloride concentration to an eluent B (sodium chloride, 2.0 M; sodium acetate buffer, 20 mM, pH 5.0). The elution was conducted at room temperature in the following sequence: (i) an initial isocratic elution with 15 column volumes (cv) of the eluent A (100%), (ii) followed by a gradient elution with 25 cv of eluent A/eluent B mixture (from 100%/0% to 75%/25%), (iii) followed by an isocratic elution with 15 cv of eluent A/eluent B mixture (75%/25%), (iv) followed by a gradient elution with 10 cv of eluent A/eluent B mixture (from 75%/25% to 0%/100%) and (v) completed by an isocratic elution with 7 cv of eluent B (100%). It should be mentioned that significant changes in sodium chloride concentration, during the gradient elution can affect the baseline calibration of certain MALS detectors, introducing errors to the calculations of the radius of gyration (R_g_). This issue could be alleviated with the use of multiple angles MALS detectors. Optilab T-rEX detector allows effective RI detection up to a sodium chloride concentration of 0.5 M, above which (at higher conductivity), RI signal could not be properly detected in our system.

### Cytopathic effects of the ulvan extracts in vero E6 cell assay for SARS-CoV-2

#### Cytopathic effect (CPE) assay with vero E6 cells

The assay was performed by the Southern Research (Birmingham, AL) High-Throughput Screening Center. Mammalian Vero E6 cells selected for this CPE assay were capable of expression of the angiotensin-converting enzyme 2 (ACE2) receptor to which SARS-CoV-2 typically binds (Lan et al., 2020). The Vero E6 cells were obtained from Dr. Ralph Baric at the University of North Carolina. Vero E6 cells were grown in Minimum Essential Media (MEM) supplemented with 10% of Heat-Inactivated Fetal Bovine Serum (HI FBS). On the day of assay, the cells were harvested with MEM supplemented with 1% Pen/Strep and 2% HI FBS. Subsequently, the harvested cells were batch inoculated with SARS-CoV-2 virus (strain USA-WA12020) having a Multiplicity of Infection (MOI) ratio of 0.002. This inoculation resulted in 5% of cells’ post-infection viability after 72 h. Assay Ready Plates (ARPs, 384-well plate, Corning 3712BC) were prepared in a BioSafety Laboratory level 2 (BSL-2) facility, by adding 5 µL of analyzed samples to the ARPs. In control wells, only MEM supplemented with HI FBS (5 µL) was added. Then, these ARPs were transferred to a BioSafety Laboratory level 3 (BSL-3) facility, where 25 µL aliquots of SARS-CoV-2 virus inoculated cells were added to each well (4,000 Vero E6 cells per well), bringing the total volume per well of 30 µL. After incubating ARPs at 37 °C/5% CO_2_ and 90% humidity for 72 h, 30 µL of Cell Titer-Glo (Promega) was added to each well. To measure cells’ viability, following incubation at room temperature for 10 min., the luminescence was measured by using a plate reader (Perkin Elmer Envision). Positive control compounds, with known *in vitro* anti-viral effects against SARS-CoV-2, were also tested in this assay. These reference compounds included Calpain Inhibitor IV, chloroquine, Remdesivir, hydroxychloroquine, and Aloxistatin (E64d).

#### Sample preparation for the CPE assay

A solid sample of ulvan extract was resuspended in MEM supplemented with 2% HI FBS solution to a concentration of 30 mg mL^−1^. It was then kept for 10 min at RT, centrifuged (13,000 rpm), and filtered through a 0.22 µm filter. The resulted stock solution was used for the preparation of the diluted samples. Subsequently, a series of dilutions was performed in which the stock solution was diluted 3-fold (per dilution) 9 times, providing 10 different concentrations of an extract in a range between 30 mg mL^−1^ and 1.52 µg mL^−1^. In the ARPs, after the addition of the cells’ solutions in media (25 µL), these 10 different concentrations were further diluted 6-fold, making the final concentration range of the assayed ulvan extract between 5 mg mL^−1^ to 0.25 µg mL^−1^. This specific concentration range was chosen to encompass both the cytotoxicity level (in a range of mg mL^−1^) and potential acute anti-viral activity (in a range of µg mL^−1^), as was reported for assays of other viruses (Witvrouw & De Clercq, 1997; Damonte, Matulewicz & Cerezo, 2004).

### Measurement of cytotoxicity effect of ulvan extracts

For measurement of the cytotoxicity effect of various ulvan extracts, the method for sample preparation and assay included all the above-described steps, except for the step in which the virus was inoculated. The cytotoxicity of different ulvan extracts was evaluated by adding 25 µL aliquot of cells, without viruses, to 5 µL of ulvan extracts and to control wells containing only cell media. After incubating these ARPs at 37 °C/5% CO_2_ under 90% humidity for 72 h, 30 µL of Cell Titer-Glo (Promega) was added to each well. To measure cells’ viability, following incubation at room temperature for 10 min., the luminescence was measured by using a plate reader (BMG CLARIOstar). For each plate, positive control with known *in vitro* cytotoxic effect against Vero E6 cells (*N*-benzyl-*N, N*-dimethyl-2-{2-[4-(2,4,4-trimethyl-pentan-2-yl)phenoxy]-ethoxy}ethanaminium chloride; hyaline, 100 µM) was added. Each analysis was performed in two duplicate wells.

#### Assays’ output processing

Data from the plate reader were normalized to the average signal obtained from wells containing uninfected cells (*Avg Cells*, Eq. (1)), corresponding to 100% virus inhibition result. The average signal obtained from wells containing virus-infected cells (*Avg Virus*, Eq. (1)) is corresponding to a 0% virus inhibition result (wells to which no ulvan extracts or any other reference antiviral compounds were added). The tested compound (*Test Cmpd*, Eq. (1)) parameter is defined as the average signal obtained from the wells containing a sample of analyzed ulvan extract. To calculate [*% Inhibition* ] values in the CPE assay, we used, Eq. (1):

(1) \begin{eqnarray*}\text{%}\text{Inhibition}=100\times \frac{(\text{Test Cmpd}-\text{Avg Virus})}{(\text{Avg Cells}-\text{Avg Virus})} \end{eqnarray*}

The output signal which is coming from the wells containing only the Vero E6 cells is defined as the highest output signal (*Control*; Eq. (2)), corresponding to the 100% cell viability. The output signal coming from the wells containing the hyamine-treated cells is defined as the lowest output signal (*Avg Hyamine*; Eq. (2)), corresponding to the 0% cell viability and the highest cytotoxic effect in this assay. To calculate [*% Cell Viability* ] values in the cytotoxicity assay, we used Eq. (2):

(2) \begin{eqnarray*}\text{%}\text{Cell Viability}=100\times \frac{(\text{Test Cmpd}-\text{Avg Hyamine})}{(\text{Control}-\text{Avg Hyamine})} \end{eqnarray*}

The values of the half-maximum viral inhibitory concentration (IC_50_) and the half-maximum cytotoxicity concentration (CC_50_) were obtained by using non-linear regression, fitting the concentration–response titration data into 4-parameters Hill equation, allowing us to determine the IC_50_, CC_50_, and the Selectivity Index (SI = CC_50_/IC_50_) values for AOx and HCl ulvan extracts.

## Results

Our project focused on the cultivation, extraction, bioassay, and chemical analysis of green seaweed *Ulva* sp. ulvan extracts. The lab cultivation was preferred to reduce to the minimum various contaminations in the *Ulva* sp. biomass, which may come from its native marine habitat. By controlling the light exposure, nutrients composition, aeration, and temperature, the lab cultivation of the *Ulva* sp. could allow for better control over variability in the resulted biomass composition in future commercial production.

### Bioassay of extracts

In this study, we used a Cytopathic Effect (CPE) reduction assay, performed with mammalian Vero E6 cells that were infected with the SARS-CoV-2 virus. This type of essay is popular and widely used for screening agents for their antiviral activity (Heaton, 2017). CPE assay allows to correctly evaluate the broad antiviral activity potential of tested material, regardless of the inhibited infection stage, such as virus binding to host cell receptor, entering the host cells, replication, assembly, budding, and reinfection of neighboring cells. In our CPE assay, the mammalian Vero E6 host cells, capable of expressing the angiotensin-converting enzyme 2 (ACE2) receptor, to which SARS-CoV-2 typically bind, were inoculated with this virus. The virus utilizes the host cell machinery for its replication and spreading, ultimately leading to the death of the infected cell. Efficient antivirals are not cytotoxic and are capable of maintaining the viability of the SARS-CoV-2 inoculated cells from the cytopathic effect of the virus. For the CPE assay, Vero E6 cells inoculated with SARS-CoV-2 virus were grown in media containing various concentrations of the tested compounds. The cell viability was assessed after 72 h of incubation. The assay boundaries were the viability of the uninoculated Vero E6 cells, which represent 100% inhibition of the virus activity, and untreated cells inoculated with the SARS-CoV-2 virus, which represents 0% inhibition (virus-induced cell’s death). To test the cytotoxicity of our ulvan extracts against Vero E6 cells, we compared them, in the absence of SARS-CoV-2 virus, to a known cytotoxic reference compound –*N*-benzyl-*N, N*-dimethyl-2-{2-[4-(2,4,4-trimethyl-pentan-2-yl)phenoxy]-ethoxy}ethanaminium chloride (hyamine). The activity of *Ulva* sp. crude ulvan extracts was compared to a series of reference synthetic antivirals, which were shown to be active against SARS-CoV-2 virus *in vitro*, including Calpain Inhibitor IV, chloroquine, Remdesivir, hydroxychloroquine, and Aloxistatin (E64d) (Table 1) (Chen et al., 2020a).

**Table 1:** Summary of CPE assay with Vero E6 host cells for anti-SARS-CoV-2 ulvans crude extract activity.

**Compound**	**Activity against** **SARS-CoV-2**	**Max% inhibition**	**IC**_**50**_	**CC**_**50**_	**Cytotoxic activity**	**Min% viability**
Ulvan (by HCl Protocol)	Inactive	6.65	>5.00 mg mL^−1^	3.75 mg mL^−1^	Active	38.17
Ulvan (by AOx Protocol)	**Active**	75.28	4.14 mg mL^−1^	3.58 mg mL^−1^	**Active**	50.81
Calpain Inhibitor IV	Active	113.79	0.131 µg mL^−1^	>4.00 µg mL^−1^	Inactive	95.21
Chloroquine	Active	103.85	1.184 µg mL^−1^	>9.60 µg mL^−1^	Inactive	93.95
Remdesivir	Active	103.02	2.944 µg mL^−1^	>18.08 µg mL^−1^	Inactive	92.7
Hydroxychloroquine	Active	101.33	1.865 µg mL^−1^	>10.08 µg mL^−1^	Inactive	94.19
Aloxistatin (E64d)	Active	74.58	4.908 µg mL^−1^	>10.27 µg mL^−1^	Inactive	92.78

**Figure 1:**
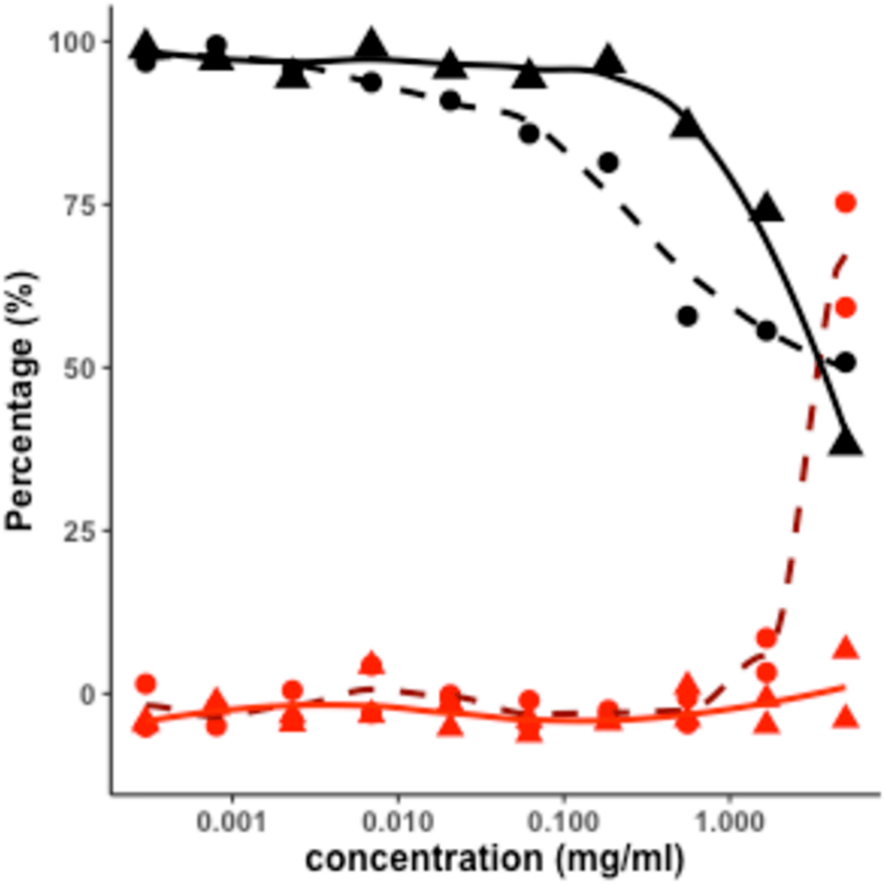
SARS-CoV-2 infection inhibition in Vero E6 cells with ulvan. Percentage of inhibition of SARS-CoV-2 virus in Vero E6 cells in the CPE assay, *Ulva sp.* extracts: by AOx Protocol (dashed dark red curve, with • dots); by HCl Protocol (solid red curve, with ▴ dots). Percentage of the viability of the Vero E6 cells in the cytotoxicity assay, of *Ulva sp.* extracts: by AOx Protocol (dashed black curve, with • dots); by HCl Protocol (solid black curve, with ▴ dots). For each concentration, measured in duplicates, curves were drawn using Locally Estimated Scatterplot Smoothing methodology.

We found that AOx ulvan extract showed anti-SARS-CoV-2 positive activity (IC_50_ 4.14 mg mL^−1^) with some cytotoxic effects (CC_50_ 3.58 mg mL^−1^) (Table 1, Fig. 1). The Vero E6 cells viability in the AOx ulvan extract experiment was about 70%, which is 14 times higher than the same cell viability observed in the negative control experiment, which included inoculation with the virus. In contrast, even at its maximum tested concentration (5.00 mg mL), the HCl-based extract did not show any inhibition of the SARS-CoV-2 virus in the CPE assay, while having the same cytotoxic activity (3.58 mg mL^−1^) as the AOx-based extract.

These data show that the AOx-based extract exhibited apparent anti-viral activity against SARS CoV-2, albeit only at the highest permissible concentration (5 mg/ml) tested in the assay, achieving a 75% reduction of the virus-induced cytopathic effect. At this same concentration, the AOx-extract reduced the viability of uninfected host cells to 50% of that measured in untreated control wells. Thus, the reduction in virus-induced CPE may be a consequence of the effect of the extract on the health of host cells, compromising their ability to support viral replication. However, the observation that the HCl extract exhibited a similar cytotoxic effect on the host cell could not yet reduce CPE suggests that the cytotoxic effect *per se* is not the reason for CPE reduction.

We suggest that the inhibition of SARS-CoV-2 induced CPE in Vero E6 cells is due to an antiviral activity of the AOx-based extract. Whether this effect results from direct action on the virus or is mediated through a host cell target needs to be elucidated in follow-up studies. Regarding a host target, it will be necessary to evaluate antiviral activity in assays using cells from the human lung which is a major site of virus infection. In addition, an assay with TMPRSS2 should be used for further screening. To the best of our knowledge, this is the first report of *Ulva* sp.-derived crude extract bioactivity against the SARS-CoV-2. Although the *in vitro* potency of the AOx-based extract in this assay is much lower (IC50 = 4.14 mg mL^−1^) compared to the small organic antiviral molecules tested in parallel (IC50 = 4.00 to 18.08 µg mL^−1^; Table 1), the active component(s) of the AOx-extract needs to be purified and might be more potent in assays that directly measure virus load and replication (Guo et al., 2019; Gorshkov et al., 2021).

### Chemical characterization of extracts

**Figure 2:**
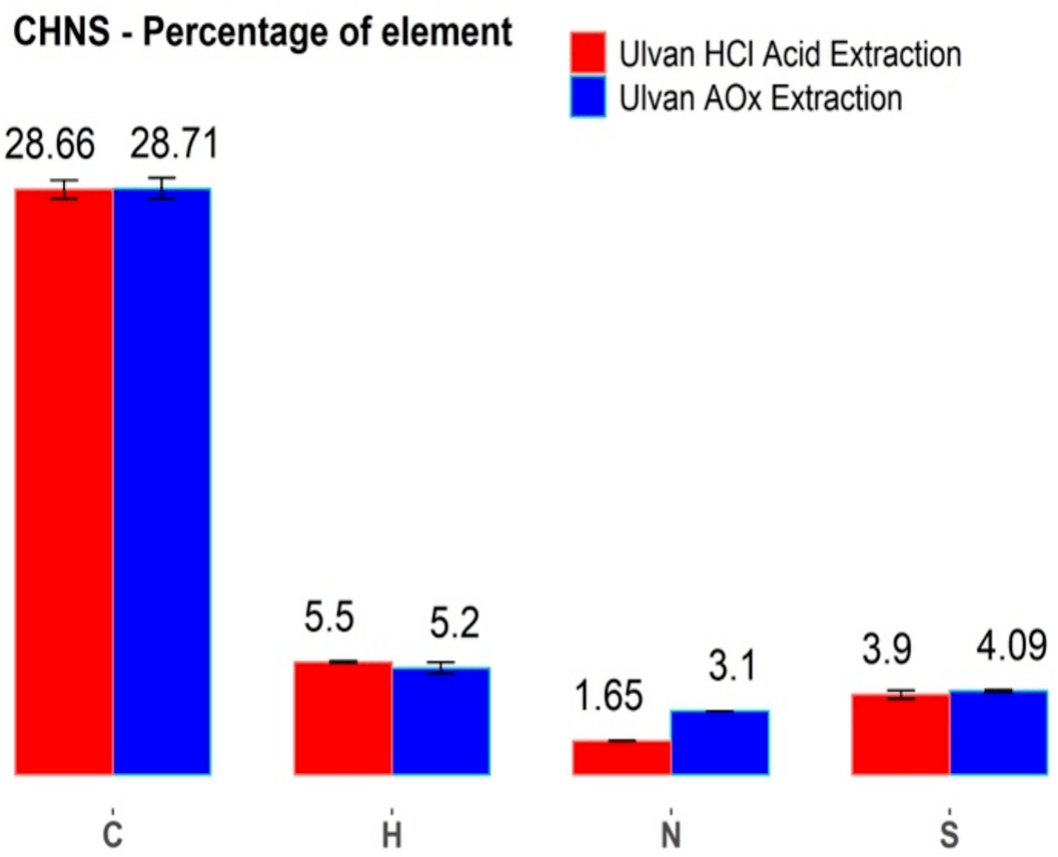
Ulvan elemental analysis. CHNS elemental analysis of the two *Ulva* sp. ulvan extracts. (*red columns*): extraction by HCl protocol and (blue columns): extraction by AOx protocol.

In an attempt to find differences in the chemical composition of HCl- and AOx-based ulvan extracts, we analyzed these materials by combustion elemental analysis of carbon, hydrogen, nitrogen, and sulfur elements (CHNS analysis, Fig. 2). The results of the CHNS analysis of materials obtained by both extraction protocols showed a close content similarity for carbon, hydrogen, and sulfur elements. The main difference between the two types of extracts was observed in the nitrogen content of the resulted materials, which was 88% higher in the case of AOx-based protocol. This result suggests a higher protein content in the AOx protocol-derived material, indicating that under neutral pH conditions of the AOx extraction more proteins underwent co-extraction with sulfated polysaccharides (Kidgell et al., 2019; Glasson et al., 2017; Wahlström et al., 2020). In the case of the HCl-based protocol, at acid pH conditions most of the proteins are found to be poorly soluble and thus remain in the solid residues (Kidgell et al., 2019). Besides, it is plausible that a higher degree of protein hydrolysis to amino acids and peptides also took place at pH 2 and 75 °C. These low MW compounds were subsequently removed by dialysis during the purification of the extracts.

The sulfur content in both types of extracts is important, as most of the previous literature regarding the antiviral activity of seaweed sulfated polysaccharides showed a good correlation between the higher sulfur content of the evaluated material and its higher bioactivity (Witvrouw & De Clercq, 1997; Ghosh et al., 2009; Chen et al., 2020b; Damonte, Matulewicz & Cerezo, 2004). Since in our case, the sulfur content in both types of extracts was practically identical (Fig. 2), a possible antiviral activity of the AOx extract may come from differences in the polysaccharides’ structures and/or due to the formation of protein- polysaccharides complexes and aggregates.

**Figure 3:**
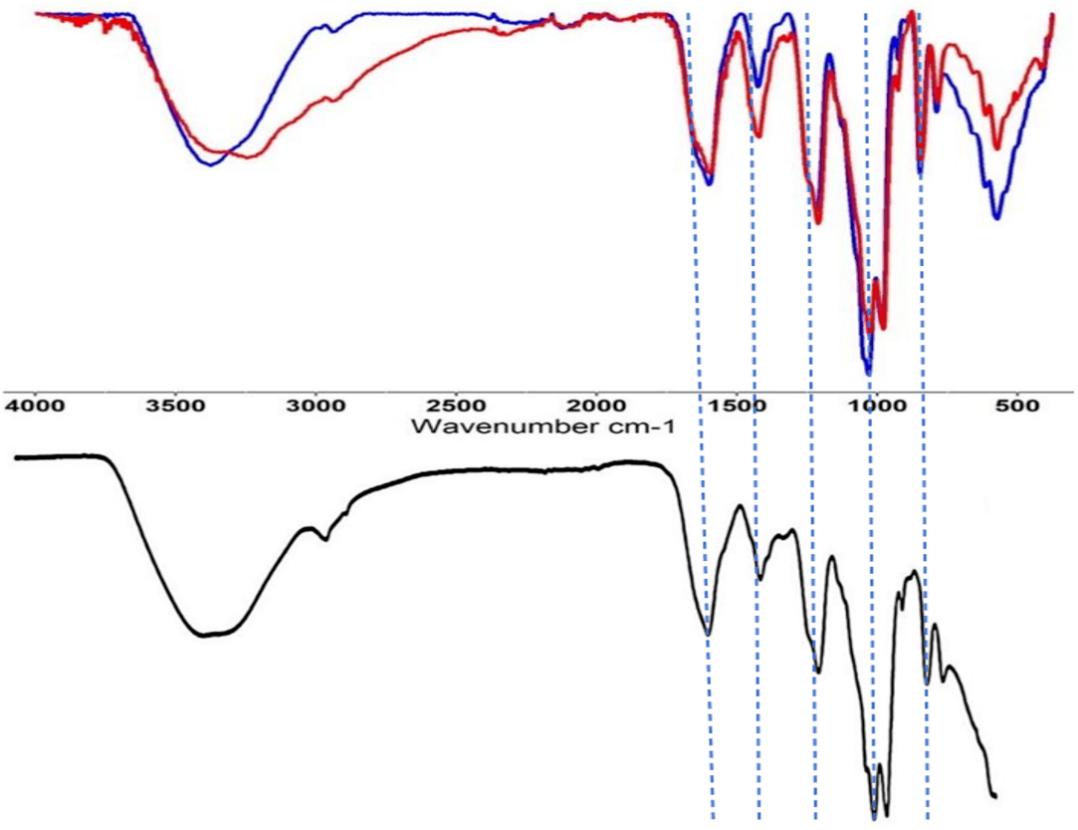
Fourier-transform infrared spectroscopy (FTIR) of ulvans. FTIR spectra of extracted ulvan from *Ulva* sp. Top spectra: (blue spectrum): material obtained in HCl-based extraction; (red spectrum): material obtained in ammonium oxalate-based extraction. Bottom spectra: reference spectra of ulvan from Ulva armoricana, adapted from Wahlström et al. (2020).

In addition to the elemental analysis, both extracts (in their lyophilized form) were analyzed by the attenuated total reflection Fourier transform infrared spectroscopy (ATR-FTIR; Fig. 3). The FTIR spectra of materials obtained from HCl- and AOx-based extraction were quite typical to the spectra of ulvans extracted from different species of Ulva *sp*. and by different extraction protocols (Wahlström et al., 2020).

Both measured FTIR spectra for the HCl- and AOx-protocols exhibited a high degree of similarity, especially for the following peaks: 848 cm^−1^ (corresponding to the stretching of C-O-S bonds, usually found in ulvan, due to the presence of the sulfate groups), 983 cm^−1^ (corresponding to the stretching of C-O bonds in sugars), 1,215 cm^−1^ (corresponding to the stretching of S=O bond of the sulfate groups) and 1,600 cm^−1^ (corresponding to the carboxylic groups of the uronic acid moieties). Major differences between our FTIR spectra were found in the intensities of the peaks, which were stronger for the material produced by the HCl protocol. These include peaks at 573 cm^−1^, 1,032 cm^−1^ (corresponding to the symmetric stretching of C-O-C bonds of carboxylic groups), 1,425 cm^−1^ (corresponding to the asymmetric stretching of O-C-O bonds of carboxylic groups), 2,940 cm^−1^ (corresponding to the stretching of C-H bond) and around 3,375 cm^−1^ (corresponding to the O-H stretching of the hydroxyl groups). Only small differences were present between the FTIR spectra of the HCl and AOx extracts, and it was found to be closely similar to the FTIR spectra of a reference commercial “winter-heavy” ulvan (by CarboSynth, UK) extracted from *Ulva armoricana* collected in Bretagne, France (Wahlström et al., 2020). Overall, all our extracted ulvans showed similar absorbance profiles to previously published FTIR spectra of ulvan (Robic et al., 2009a).

### Fraction analysis of the extracts

Generally, ulvan extracts contain branched polysaccharides with a broad distribution in terms of their charge density and MW (Kidgell et al., 2019; Wahlström et al., 2020; Robic et al., 2009a; Robic et al, 2009b). Also, ulvan extracts can contain minor quantities of proteins, nucleic acids, phenolic compounds, and metal salts. For a better characterization of our extracts, we performed chromatographic separation by mass / hydrodynamic radius using Size Exclusion Chromatography (SEC), or by charge using Anion Exchange Chromatography (AEIX). The elution was monitored by a Diode-Array UV detector, coupled with multi-angle light scattering (MALS) and Refraction Index (RI) detectors.

### SEC analysis

**Figure 4:**
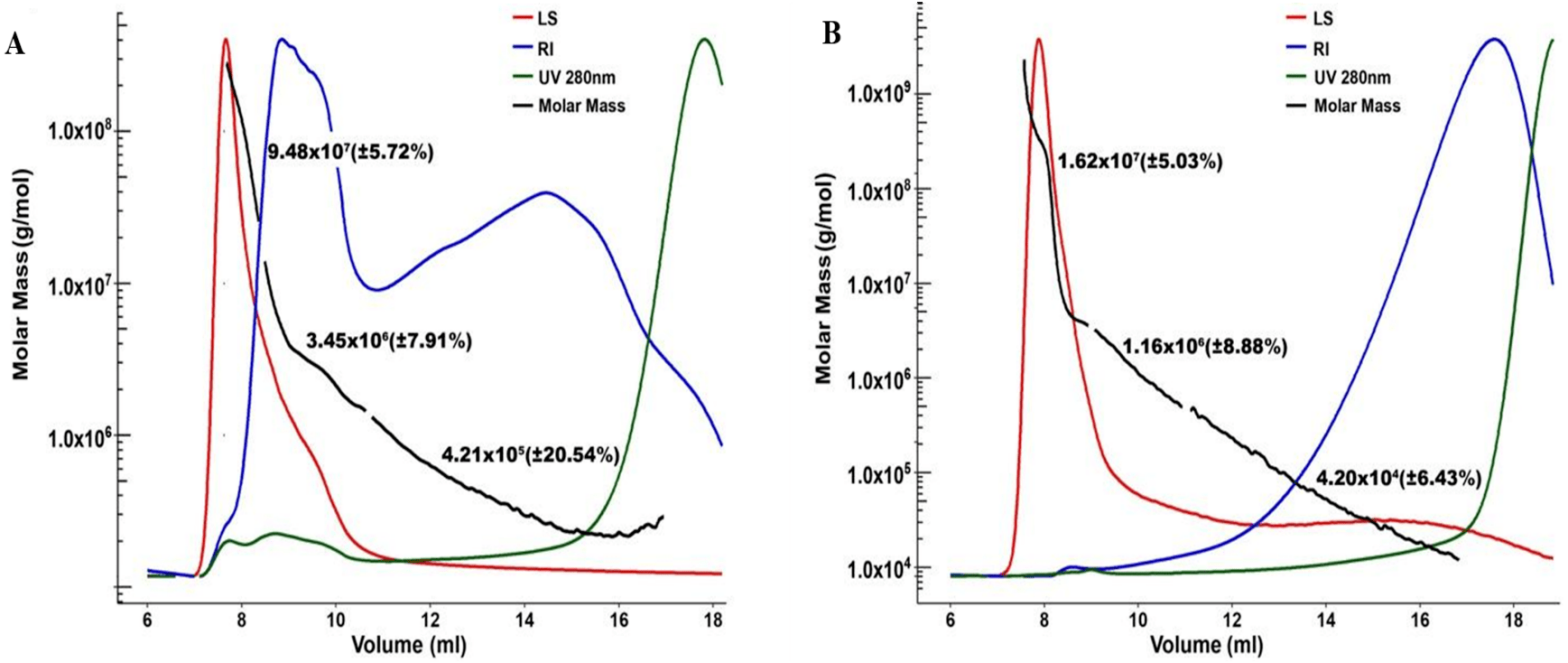
SEC-MALS chromatograms in Superose of AOx. (A) and HCl (B) extracts monitored by UV absorbance detector at 280 nm (green chromatogram), light scattering detector (LS, red chromatogram), Refractive Index detector (RI, blue chromatogram) detector and by MALS detector (average Molar Mass, black elution curve) (A, B).

SEC-analysis of the two crude ulvan extracts showed significant differences in composition (Fig. 4). Absorbance at 280 nm in both samples of AOx (Fig. 4A) and HCl extracts (Fig. 4B), indicated the presence of proteins in the analyzed samples. This absorbance was detected throughout the entire sample elution, starting from the void volume (minor quantities) and gradually increasing until the end of the elution (larger quantities). However, the absorbance at 280 nm in the chromatogram of the AOx extract exhibited two unseparated peaks eluting between 7 to 11 mL, which were practically not present in the chromatogram of the HCl extract.

The chromatogram of the AOx extract monitored by the RI detector exhibited a profile with two unseparated peaks, where the higher peak eluted between 7 to 11 mL elution volumes, and the smaller one eluted between 11 to 18 mL elution volumes. The chromatogram of the HCl extract monitored by the RI detector showed a different profile, with a unique broad peak eluting between 12 to 18 mL elution volumes. These two chromatogram profiles indicated the presence of a wide distribution of MW and/or chemical compositions in the polysaccharide extracts. They also revealed a profound difference in the composition of the two extracts. Each of the chromatograms of the two extracts, monitored by the LS detector, showed a strong peak eluting between 7 to 11 mL elution volumes. It could be attributed to the higher sensitivity of such detector to high MW polysaccharides in comparison to low MW polysaccharides, even if the latter is in higher concentration.

Overall, higher signals as monitored by the LS detector were observed in the chromatogram of the AOx extract than in the chromatogram of the HCl extract. The LS/RI profile indicated the presence of branched polysaccharides with a broad distribution of MW in the fractionation range of the SEC analytical column (Superose 6 Increase), i.e., from 5 to 5,000 kDa. Three populations of average MWs could be distinguished in each SEC chromatogram (black curves in Figs. 4A and 4B). More specifically, we are referring to the fractions with an average molecular weight of 9.48 × 10^7^ and 1.62 × 10^7^ g moL^−1^ for the first eluting fraction (between 7 to nine mL elution volumes), 3.45 × 10^6^ and 1.16 × 10^6^ g moL^−1^ for the second eluting fraction (between 9 to 11 mL elution volumes), and 4.21 × 10^5^ and 4.20 × 10^4^ g moL^−1^ for the third eluting fraction (between 11 to 17 mL elution volumes), for the AOx and HCl extracts, respectively. For all three populations, the average MWs were significantly higher in the AOx extract (Fig. 4A) than in the HCl extract (Fig. 4B). Extraction at acidic conditions at a temperature above 70 °C seemed to induce minor hydrolysis of the extracted SSPS leading to a corresponding minor reduction in the obtained average MWs by SEC compared to the extraction at neutral pH (AOx protocol). Those results are consistent with previous reports that acid extraction protocols yield lower MW polysaccharides than with other ammonium oxalate-based protocols (Kidgell et al., 2019; Robic et al., 2009a; Robic et al, 2009b). Calculations of the average MWs by the software (ASTRA) should be analyzed with the recognition of the limitations of the light-scattering detector utilized in the present work. The latter is a tri-angle light scattering detector, which is not as accurate as a multi-angle detector for measuring the MW of mixtures with a complex chemical composition including branched polymers. It is noteworthy to mention that the void volume of the used SEC column is around seven mL, its total volume is 23 mL, and what is eluting after 18 mL are very low MW molecules that are below the fractionation range of the column. Thus, the peaks monitored by the RI and UV detectors after the elution of 18 mL are referring to materials with an average MW below 5 kDa and therefore not relevant for the SEC analysis of our polysaccharides of interest. The analysis by SEC revealed differences in the MW distribution in the two extracts. The difference in chemical composition between the two extracts was then investigated using AEX, which is a more suitable tool for the fractionation of mixtures of charged polymers.

### AEX analysis

**Figure 5:**
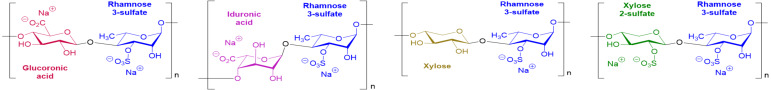
Structures of the main disaccharide units present in sulfated polysaccharides from *Ulva* sp. (Kidgell et al., 2019).

Ulvan contains two types of negatively charged groups: sulfate esters (on rhamnose and xylose moieties) and carboxylate groups (glucuronic or iduronic acids moieties) that are distributed in repeating disaccharides structures, as described in Fig. 5 (Kidgell et al., 2019; Wahlström et al., 2020). The presence of these charged groups allows the fractionation of the two SPSS extracts by the anion-exchange chromatography. For our extracts, a relatively low pH (5.0) was used to reduce the strong binding of the negatively charged polysaccharides to the used AEX column. Fractionation of Ulvan extracts was achieved by using a strong anion exchange Mono-Q column, utilizing eluent with a gradually increased concentration of sodium chloride (from 0 mM to 2 M) in sodium acetate buffer (20 mM, pH 5.0).

**Figure 6:**
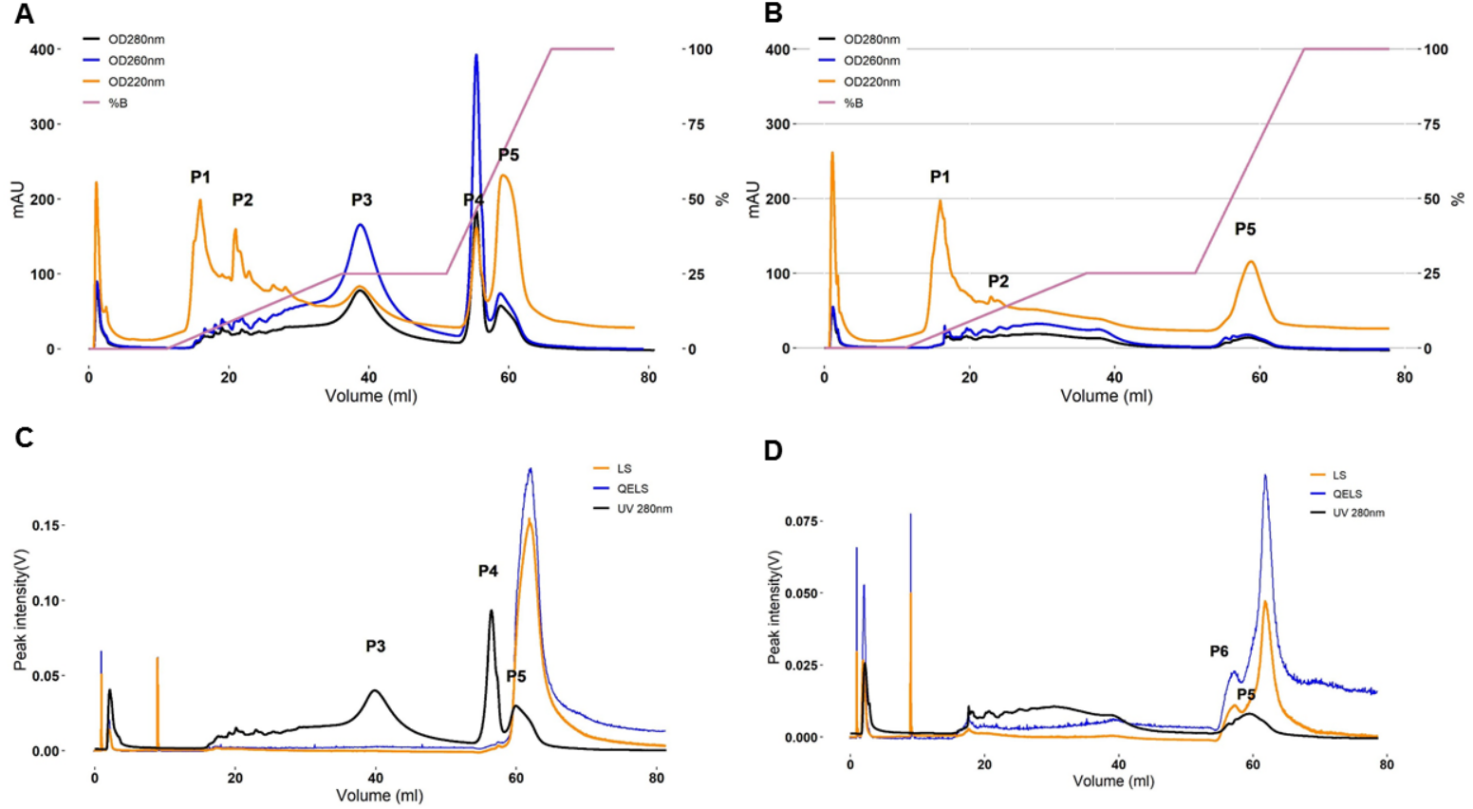
AEX-MALS chromatograms of ulvan extracts. AEX-MALS chromatograms of ulvan extracts from *Ulva sp*. using the AOx (A, C) and HCl (B, D) protocols, respectively. UV absorbance detector monitored at three wavelengths of 280 (blue chromatogram), 260 (red chromatogram), and 220 nm (pink chromatogram) (A, B). Chromatograms of the UV absorbance at 280 nm (green chromatogram), light scattering (LS, red) detector, and quasi-elastic light scattering (QELS, blue) detector are shown in C & D for the AOx, and HCl, protocols, respectively. P1 to P6 correspond to six different elution peaks.

AEX chromatograms of SSPS extract from *Ulva* sp., obtained from AOx (Fig. 6A) and HCl protocols (Fig. 6B), respectively, show substantial differences in the composition of the extracted materials. As monitored by the UV detector at 220 nm, the main eluted peaks which are corresponding to 5 different fractions of organic materials in these extracts, were identified according to their volume of elution and named in their order of elution: from P1 to P5 (Fig. 6). The first peak (P1, Figs. 6A and 6B) was eluted at 16 mL (at the concentration of NaCl of 28 mM) and corresponded to the elution of the void volume.

Notably, the AEX chromatogram monitored by the UV detector at 260 and 280 nm of the AOx extract showed apparent nucleic acids/proteins peaks (with a prevalence of the former as suggested by the ratio of the absorbance at 260 and 280 nm) at around an elution volume of 20 mL, corresponding to the eluting peaks between P1 and P2 (Fig. 6A) at the gradient condition of NaCl of about 110 mM, at 39 mL (P3, NaCl 480 mM), 55.5 mL (P4, NaCl 800 mM), and at 58.5 mL (P5, ∼1 M NaCl). In comparison, the number of proteins and nucleic acids was much lower in the chromatogram of the HCl extract, with a small wide peak eluting from 19.6 mL (NaCl ∼100 mM) to 39 mL (NaCl 480 mM) and a second narrower peak at 55.5 mL (P5, NaCl ∼800 mM) (Fig. 6B).

Analyzing signals from the UV detector at 220, 260, and 280 nm, we concluded that the peaks P3 and P4 could be attributed to proteins and nucleic acid contaminants. Those two peaks were absent in the chromatogram of the HCl extracts and we can conclude that the HCl protocol yielded an extract of SSPS with higher purity, in comparison to the AOx extraction protocol. These results were in line with the higher nitrogen content observed in the abovementioned results of CHNS analysis and with previous reports comparing the extraction of SSPS from *Ulva* sp. biomass with similar protocols (Kidgell et al., 2019). Thus, only the materials eluted at peaks P1, P2, and P5 are of interest, as their UV signal corresponded to potential fractions of polysaccharides.

The monitoring of UV absorbance at 280 nm and LS and Dynamic Light Scattering (QELS) signals during the analysis are showed for the AOx (Fig. 6C) and HCl (Fig. 6D) extracts, respectively. Nevertheless, the peaks in the LS chromatogram showed the presence of high MW polysaccharides, eluting from 58 mL at NaCl concentration of 1 M, in both extracts; although the intensity of the signal was much higher in the chromatogram of the AOx extract in comparison to the HCl extract. Those MW polysaccharides that were tightly bonded to the AEX column seemed to refer to the targeted sulfated polysaccharides ulvan in the two extracts.

We hypothesize that P1 and P2 peaks correspond to low MW polysaccharides (eluting with other contaminants) that are not retained on the column, nor detected by the LS detector. Only P5 peak was observed in the chromatogram of the AOx extract (Fig. 6C), while in the chromatogram of the HCl extract a small additional peak shoulder P6 was visible (at a similar elution volume of P4), before the elution of the main P5 peak (Fig. 6D). The latter two peaks indicated the presence of two different populations of SSPS that could be separated by their different interactions with the AEX column, where the shoulder peak has fewer negatively charged groups than in the main peak P5. In comparison, only P5 could be observed in the chromatograms of the AOx extract as monitored by the LS and QELS detector (Fig. 6C). The peak P4 is present on the UV absorbance chromatograms but not on the LS and QELS chromatograms. This result suggests that the composition of the materials eluting at the volume corresponding to P4 and P6 is different. Overall, based on SEC and AEX analyses, the HCl extraction protocol produced more homogeneous material. However, the amount of the latter material was smaller than in the case of more heterogeneous mixture l obtained by the AOx extraction protocol.

## Discussion

Based on the CHNS, FTIR, SEC, and AEX analyses, we found that the AOx and HCl extraction protocols of *Ulva* sp. resulted in mixtures of compounds having a different distribution of molecular weights, overall molecular charges, and contaminations level with nitrogen-containing molecules. Remarkably, both these extracts had also somewhat different antiviral activity, confirming our initial hypothesis regarding the variability in the biological activity of SSPS compounds obtained by different extraction protocols. Our idea for testing the SSPS of the green seaweed *Ulva* sp. as a potential candidate against SARS-CoV-2 was based on previously reported antiviral activities of SSPS (Jin et al., 2020; Song et al., 2020; Kwon et al., 2020). In this work, we show that the AOx extracts indeed showed a positive *in vitro* antiviral activity, protecting VERO E6 cells against the cytopathic effect of the SARS-CoV-2. Our observations are in line with existing hypotheses that the antiviral activity of SSPS against SARS-CoV-2 and other viruses could be attributed to the interaction of the negatively charged groups of SSPS polysaccharides with proteins located on the envelope of viruses (Ghosh et al., 2009; Song et al., 2020; Damonte, Matulewicz & Cerezo, 2004). These interactions could be also influenced by charge density on SSPS biopolymers, their MWs, and the flexibility of the polysaccharide backbone, as we found that AOx-based extract had a higher average MW (*versus* HCl-based extract), a higher overall charge, and a more potent antiviral activity. We should mention that the IC_50_ of the AOx-based extract was in the concentration range of a few mg mL^−1^, while previously reported antiviral activity of ulvan was in the range of tens of µg mL^−1^ (Chiu et al., 2012; Lee et al., 2004; Lopes et al., 2017).

Our results indicate that either the activity of the AOx-based extract against SARS-CoV-2 was not as potent as other SSPS against other viruses, or, most probably, only a specific fraction of this AOx-based extract is active against SARS-CoV-2. The latter possibility is strongly supported by reports regarding various fractions isolated from SSPS extracts exhibiting significantly different antiviral activities (Lee et al., 2004; Lopes et al., 2017). Since the cytotoxicity of compounds obtained in the AOx- and HCl-based extraction protocols were closely comparable, yet, had an 11.3-fold difference in their maximum inhibition activity against SARS-CoV-2, it is reasonable to conclude that the antiviral activity of the AOx-based extracts may originate from a specific type of compounds found in sufficient quantity in the latter extract, but not present in the HCl-based extracts. In line with this conclusion, we found that the AOx-based extracts also exhibited a higher level of nitrogen-containing compounds, in comparison to the HCl-based extracts. Although the presence of nitrogen-containing molecules in our extraction protocols was minimized by dialysis and other purification steps, nitrogen-containing molecules could be still present in our extracts due to their strong affinity to the SSPS (Kidgell et al., 2019; Robic et al., 2009c). These nitrogen-containing molecules could be secondary metabolites, such as alkaloids, various peptides, proteins, and even fragments of nucleic acids, and they could be responsible for the difference of antiviral activity in the two extracts. For example, recently, an alkaloid caulerpin, isolated from a green seaweed *Caulerpa* sp., was predicted *in silico* to have potent anti-SARS-CoV2 activity (Ahmed et al, 2020). More interestingly, lectins, a group of carbohydrate-binding proteins with antiviral activities (including against SARS-CoV-2) are present in the *Ulva* sp. cell wall and could thus be co-extracted with the SSPS (Barre et al., 2020; Wang et al., 2004; Liu et al., 2020; Mitchell, Ramessar & O’Keefe, 2017).

Although specific isolation and precise identification of the active components in the AOx-based extract is still required, we suggest that our present work will serve as a starting point for a thorough bioassay-guided fractionation approach to identify an active fraction or active fractions combination. An additional challenge that should be addressed in future *in vivo* work is the delivery of the active fractions or crude extract. Previous studies found that polysaccharides can be used as therapeutics through the oral routes (after potential chemical modification) or by topical routes to control viral infections (Pujol et al, 2007). One of the potential pathways through which this could be achieved is using nebulized ulvan. Aerosol and spray could be used to protect the respiratory tract against contamination. For example, such an anti COVID-19 spray, containing carrageenan, a red seaweed polysaccharide, was proposed by Moakes et al. (2021). In addition, such an approach for COVID-19 patients has been proposed recently with crude nebulized heparin (van Haren et al., 2020). Yet, the process for ulvan nebulization and assessment of its bioavailability and efficacy needs further development and pre-clinical and clinical validation.

Despite worldwide vaccination campaigns, it is crucial to continue the efforts in the discovery of new antiviral therapeutic agents. One of the major reasons is the continuous appearance of new virus mutants some of which exhibiting resistance to both vaccine-based immunity and known antivirals. The second reason is related to the antiviral activity of certain SSPS, which are offering a vast chemical library and a platform for the development of broadly active antivirals, with potential high specific activity against certain viruses, including variants of known viruses.

## Conclusions

In this work, we provide experimental data regarding the inhibition of SARS-CoV-2 by ulvan crude extract. We compared the chemical composition and bioactivity of crude extracts obtained from an HCl-based and an ammonium oxalate-based (AOx) extraction protocols, using green seaweed *Ulva* sp. as a source of the SSPS ulvan. The composition of materials obtained by both extraction protocols was analyzed by infrared spectroscopy, CHNS elemental analysis, size exclusion (SEC), and ion exchange (AEC) chromatography, while the antiviral activity evaluation of these compounds was performed in a cytopathic assay on VERO E6 cells. The main conclusions of our study were that the extraction protocol had a significant impact on the chemical composition of the extracted SSPS, which included variations in molecular weight distribution, charge, and the level of nitrogen-containing contaminations. Also, there was a significant difference in the antiviral activity of the two extracts which could be due to the difference in chemical composition. The AOx-based extract was found to have higher average molecular weight, stronger charges, and higher antiviral activity *versus* HCl-based extract. Yet, the AOx-based extract had a more heterogeneous composition.

## Supplemental Information

10.7717/peerj.12398/supp-1 Supplemental Information 1 Supplementary Data

10.7717/peerj.12398/supp-2 Supplemental Information 2 CHNS raw data

10.7717/peerj.12398/supp-3 Supplemental Information 3 Calibration of Superose 6 Increase 30 × 1 cm 23 ml column with globular MW markers

10.7717/peerj.12398/supp-4 Supplemental Information 4 Raw data: AntiViral Report Method for measuring antiviral effect of compounds Vero E6 cells selected for expression of the SARS CoV receptor (ACE2; angiotensin-converting enzyme 2) were used for the CPE assay. Cells were grown in MEM/10% HI FBS supplemented and harvested in MEM supplemented 2% HI FBS. Cells were batch inoculated with SARS CoV-2 (M.O.I. 0.002) which resulted in 5% cell viability 72 h post infection. Compounds were dissolved in media at 30 mg/ml and serially diluted 3-fold in media nine times. Assay Ready Plates (ARPs; Corning 3712BC) were prepared in the BSL-2 lab by adding 5 µL appropriate sample dilution to wells in columns 3–12 (high to low concentration) as follows: Row A, compound 1; Row B; compound 2; Row C; compound 3; Row D; compound 4. The plates were passed into the BSL-3 facility where a 25 µL aliquot of virus inoculated cells (4000 Vero E6 cells/well) was added to each well in columns 3–22. The wells in columns 23–24 contained virus infected cells only (no compound treatment). Prior to virus infection, a 25 µL aliquot of cells was added to columns 1-2 of each plate for the cell only (no virus) controls. After incubating plates at 37° C/5%CO_2_ and 90% humidity for 72 h, 30 µL of Cell Titer-Glo (Promega) was added to each well. Luminescence was read using a Perkin Elmer Envision or BMG CLARIOstar plate reader following incubation at room temperature for 10 min to measure cell viability. Raw data from each test well was normalized to the average signal of non-infected cells (Avg Cells; 100% inhibition) and virus infected cells only (Avg Virus; 0% inhibition) to calculate % inhibition of CPE using the following formula: % inhibition = 100*(Test Cmpd - Avg Virus)/(Avg Cells –Avg Virus). The SARS CPE assay was conducted in BSL-3 containment with plates being sealed with a clear cover and surface decontaminated prior to luminescence reading.

10.7717/peerj.12398/supp-5 Supplemental Information 5 Raw data: CytoToxicity Report Method for measuring cytotoxic effect of compounds Compound cytotoxicity was assessed in a BSL-2 counter screen as follows: Host cells in media were added in 25 µl aliquots (4000 cells/well) to each well of assay ready plates prepared with test compounds as above. Cells only (100% viability) and cells treated with hyamine at 100 µM final concentration (0% viability) serve as the high and low signal controls, respectively, for cytotoxic effect in the assay. After incubating plates at 37° C/5%CO2 and 90% humidity for 72 h, 30 µl Cell Titer-Glo (Promega) was added to each well. Luminescence was read using a BMG PHERAstar plate reader following incubation at room temperature for 10 min to measure cell viability.

## Additional Information and Declarations

### Data Availability

The following information was supplied regarding data availability: The raw data are available as a Supplementary File.
