# Supplementary material for: Fighting SARS-CoV-2 with green seaweed Ulva sp. extract: extraction protocol predetermines crude ulvan extract anti-SARS-CoV-2 inhibition properties in in vitro Vero-E6 cells assay
Source: PeerJ. 2021 Nov 15;9:e12398. doi: 10.7717/peerj.12398 (PMC8601053; doi:10.7717/peerj.12398)
Supplement: Supplemental Information 1 [file peerj-09-12398-s001.docx]

**Supplementary Data :**

**Table S1. Calibration of Superose 6 Increase 30x1cm 23ml column with globular MW markers**

|  | **MW (kDa)** | **Vol (ml)** |
| --- | --- | --- |
| 1. | > 5000kDa | 8.35 |
| 2. | 1.340 | 11.25 |
| 3. | 880 | 12.6 |
| 4. | 670 | 12.79 |
| 5. | 440 | 14.31 |
| 6. | 134 | 15.87 |
| 7. | 67 | 17.2 |
| 8. | 32 | 18.76 |
| 9. | 13.7 | 21 |
| 10. | < 5kDa | 22.31 |
